# Stern Zero-Knowledge Identification Protocol Based on Lee Distance

**DOI:** 10.3390/e28080917

**Published:** 2026-08-15

**Authors:** Bing Liu, Xun Su, Binghong Yan, Anqi Liu

**Affiliations:** Department of Cryptography Science and Technology, Beijing Electronic Science and Technology Institute, Beijing 100070, China; 20241914@mail.besti.edu.cn (X.S.); 13293509813@163.com (B.Y.); 18315937371@163.com (A.L.)

**Keywords:** Lee metric, Stern zero-knowledge identification protocol, post-quantum cryptography

## Abstract

Post-quantum cryptography has gained urgent attention as quantum computing poses fundamental threats to traditional public-key cryptosystems. Code-based cryptography stands out as a robust post-quantum candidate, but most existing schemes are built on Hamming distance, whereas Lee distance provides a more natural error model for specific communication channels like phase-modulation channels. This paper presents the Lee–Stern zero-knowledge identification protocol, which extends the classic Stern protocol from the binary Hamming metric to the Lee metric over arbitrary prime fields. We adopt the state-of-the-art LMMT-ISD attack framework to conduct rigorous security re-evaluation and derive necessary parameter bounds for standard post-quantum security levels. Extensive experiments analyze how code length and prime modulus affect the protocol’s overheads, showing that the proposed scheme achieves equivalent security with notably shorter code length and smaller public key size than the original binary Stern protocol.

## 1. Introduction

### 1.1. Research Background and Significance

As quantum computing advances rapidly, public-key cryptosystems built on classical mathematical hardness assumptions face existential threats. Code-based cryptosystems have become important candidates for post-quantum cryptography due to their resistance to quantum computing attacks [1]. Traditional code-based cryptography mostly adopts Hamming distance, but in some specific channels such as phase modulation channels, the Lee metric provides a more natural error model for phase-modulation and similar channels [2]. At present, zero-knowledge identification protocols in the Lee metric lack systematic and complete implementations, and existing security analyses often lag behind the latest cryptanalytic techniques.

To fill this gap, we systematically investigate the Stern zero-knowledge identification protocol in the Lee metric. First, this paper extends the traditional binary field Stern protocol to prime fields $Z_{p}$ and implements a Lee–Stern protocol prototype supporting arbitrary primes p. Second, this paper overcomes the limitations of early security analyses for the Stern protocol, adopts the state-of-the-art LMMT-ISD cryptanalytic framework for the Lee metric proposed by Li and Wang, re-evaluates the security of the protocol, and derives the minimum code length and weight bounds required to achieve 128-bit post-quantum security. This research provides important theoretical basis and practical reference for the design of zero-knowledge identification schemes and digital signatures based on Lee distance in the post-quantum era.

### 1.2. Domestic and International Research Status

In 1993, Stern proposed the first zero-knowledge identification protocol based on binary linear codes, whose security is based on the syndrome decoding problem in the Hamming metric [3]. Since then, the Stern protocol has been extended to finite fields and derived various variants. These protocols can be converted into digital signature schemes through the Fiat–Shamir transform [4,5]. Lee distance was first introduced by Lee in 1958, and its coding theory has been extensively studied [2]. In cryptography, Weger et al. first extended the information set-decoding algorithm to finite field $Z_{p^{m}}$ in the Lee metric and analyzed the complexity of Prange- and Stern-type algorithms [6]. Subsequently, Li and Wang further proposed an improved ISD algorithm based on MMT technology, which significantly reduced the solution complexity of the syndrome decoding problem in the Lee metric [7]. Although the ISD attack algorithms in the Lee metric have been deeply studied, there is still no complete implementation and performance analysis of the Stern zero-knowledge identification protocol in the Lee metric [8]. In addition, existing studies mostly use earlier ISD complexity estimates when evaluating the security of zero-knowledge identification protocols [4,5], failing to obtain accurate security parameters combined with the latest attack algorithms. Additionally, recent NIST Post-Quantum Cryptography standardization candidates such as CROSS [9] and LESS [10] have successfully explored code-based zero-knowledge proofs, further highlighting the relevance of generalized metric variants in modern authentication schemes.

### 1.3. Main Contributions of This Paper

In view of the above shortcomings, this paper conducts a systematic study on the Stern zero-knowledge identification protocol in the Lee metric, with the main contributions as follows:

Extends the classic Stern protocol from binary fields $F_{2}$ to finite field $Z_{p}$ (where p is a prime), and implements a Lee–Stern protocol prototype supporting arbitrary p.

Breaks through the limitation that traditional zero-knowledge identification protocols rely on outdated complexity estimation. This paper introduces and applies the latest LMMT-ISD attack algorithm framework for the Lee metric proposed by Li and Wang [7]. Taking this cutting-edge cryptanalytic result as the evaluation benchmark, it conducts a rigorous re-evaluation of the security of the Lee–Stern protocol and derives the minimum code length and weight bounds required to meet the practical 128-bit security level.

Quantifies the impact of code length n and prime p on the computational and communication overheads of the protocol through a large number of experiments, and gives parameter selection suggestions for 128-bit security combined with security analysis.

## 2. Preliminaries

### 2.1. Lee Distance and Lee Weight

Let p be a prime, and $Z_{p}$ be the integer finite field modulo p. For $x\in Z_{p}$, its Lee value is defined as [2]:

(1) $${\left|x\right|}_{L}=min\left\{x,\;p-x\right\}$$

For a vector $x=\left(x_{1},\ldots ,x_{n}\right)\in Z_{p}^{n}$, its Lee weight is the sum of the Lee values of each component [2]:

(2) $$wt_{L}\left(x\right)=\sum_{i=1}^{n}{\left|x_{i}\right|}_{L}$$

The Lee distance between two vectors x,y is defined as $d_{L}\left(x,y\right)=wt_{L}\left(x-y\right)$. When p=2, Lee distance degenerates to Hamming distance.

A Lee sphere is defined as $S_{L}\left(n,w,p\right)=\left\{x\in Z_{p}^{n}:wt_{L}\left(x\right)=W\right\}$, and its size is denoted as $F_{L}\left(n,w,p\right)$. The exact expression of this quantity can be calculated through generating functions or recurrence formulas.

### 2.2. Syndrome Decoding Problem

In code-based cryptography, the syndrome decoding problem is the underlying core for constructing zero-knowledge proofs and public-key encryption schemes. Traditional SDP is usually defined over binary fields and adopts the Hamming metric [1]. For the purpose of introducing richer algebraic structures and adapting to specific physical channel models, this paper extends it to prime fields $Z_{p}$ and adopts the Lee metric.

Let C be a linear code defined over a prime field $Z_{p}$ with code length n and information bit dimension k. This linear code can be uniquely determined by a full-rank parity-check matrix $H\in Z_{p}^{\left(n-k\right)\times n}$. For any error vector $e\in Z_{p}^{n}$, its corresponding syndrome vector s is defined as:

(3) $$s=He^{T}\mathrm{mod}p$$

Based on the above algebraic structure, we formally define the syndrome decoding problem in the Lee metric as follows:

**Definition 1.**  *Syndrome Decoding Problem in the Lee Metric: Given a prime p, code length n, information bit dimension k, a random full-rank parity-check matrix* $H\in Z_{p}^{\left(n-k\right)\times n}$ *, a syndrome vector* $s\in Z_{p}^{n-k}$ *, and an integer weight bound* w *, find an error vector* $e\in Z_{p}^{n}$ *such that* $He^{T}=s^{T}\mathrm{mod}\;p$ *and the Lee weight of the vector satisfies* $wt_{L}\left(e\right)\leq w$ *[11].*

### 2.3. Stern Zero-Knowledge Identification Protocol

**Figure 1:**
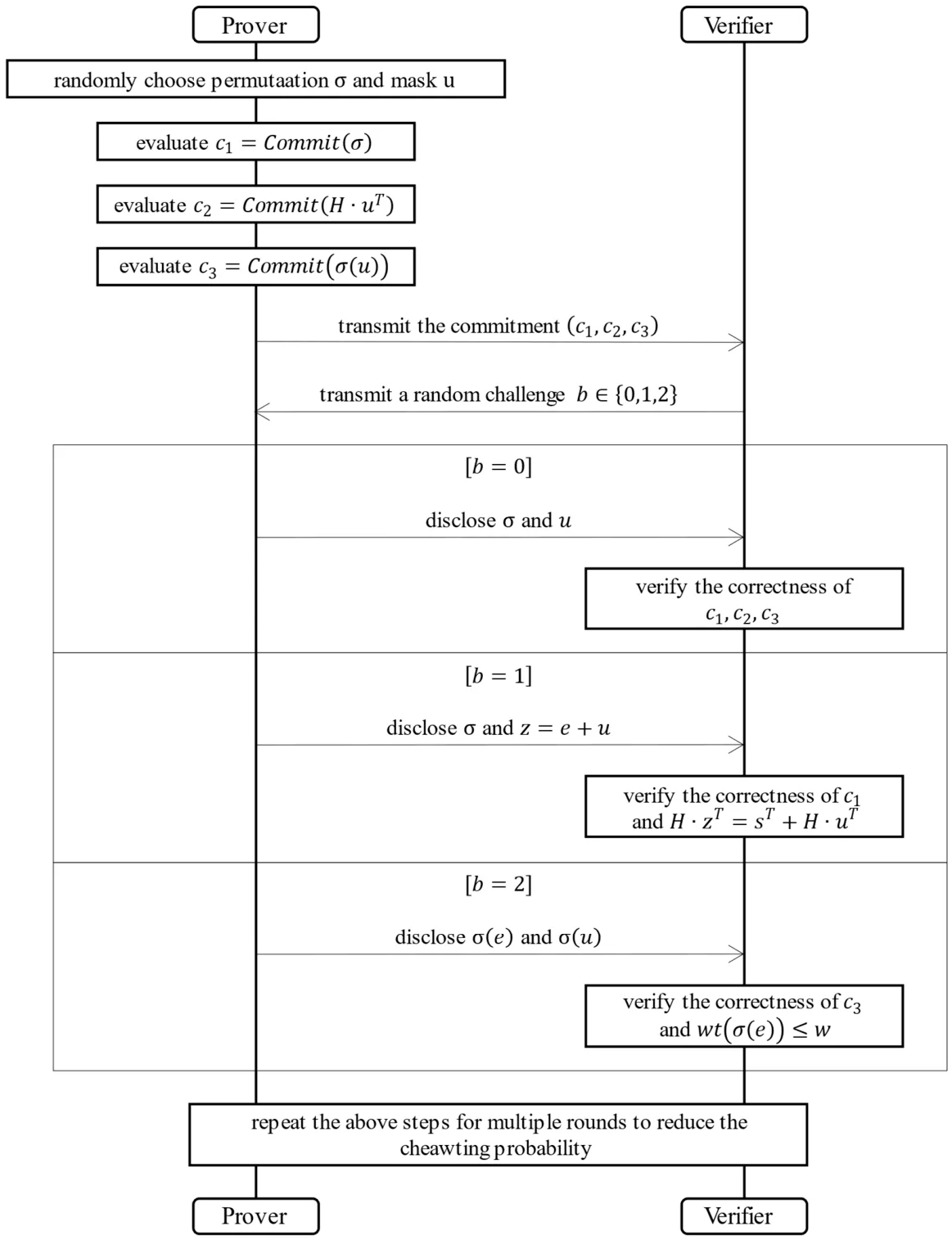
Interactive figure of the Stern zero-knowledge identification protocol.

The Stern zero-knowledge identification protocol is a three-round zero-knowledge identification scheme based on binary linear codes. The public parameters of the protocol are the parity-check matrix $H\in F_{2}^{\left(n-k\right)\times n}$, the syndrome $s\in F_{2}^{n-k}$, and the weight bound w [3]. The prover possesses a secret error vector $e\in F_{2}^{n}$ satisfying $H\cdot e^{T}=s$ and $wt_{H}\left(e\right)=w$. Each round of interaction is presented as Figure 1:

## 3. Scheme Description

### 3.1. Protocol Parameters and Key Generation

This chapter will detail the Stern zero-knowledge identification protocol based on Lee distance (referred to as the Lee–Stern protocol) proposed in this paper. We will first clarify the system parameters and key generation process of the protocol, then elaborate on the specific operation steps of the protocol in the three-round “commitment-challenge-response” interaction, and finally deeply analyze the core differences between this protocol and the classic binary Stern zero-knowledge identification protocol from two dimensions: vector space and weight calculation metric.

Before the protocol runs, the system needs to be initialized to establish public parameters, and the prover generates its public–private key pair.

**Public parameters:** Select a prime p and determine the vector space as $Z_{p}^{n}$. Set the code length n and information bit dimension k. Instead of merely bounding the Lee weight to w, we define a fixed composition (multiset) $T=\left(m_{0},m_{1},\ldots ,m_{\left\lfloor \frac{p}{2}\right\rfloor }\right)$, where $m_{i}$ denotes the exact number of elements in the error vector that have a Lee value of i. The parameters must satisfy $\sum_{i=0}^{\left\lfloor \frac{p}{2}\right\rfloor }m_{i}=n$ and the total Lee weight $w=\sum_{i=1}^{\left\lfloor \frac{p}{2}\right\rfloor }\left(i\cdot m_{i}\right)$. Let $S_{T}$ be the set of all vectors in $Z_{p}^{n}$ that have this exact composition T. In addition, select a secure cryptographic hash function. Randomly generate and disclose a full-rank parity-check matrix $H\in Z_{p}^{\left(n-k\right)\times n}$.

**Secret key:** The prover randomly and uniformly samples a vector $e\in S_{T}$. This strictly fixes the multiset of the secret values, completely avoiding the leakage of extra information during permutations. This vector e is the prover’s secret key.

**Public key:** The prover calculates the corresponding syndrome $s^{T}=He^{T}\left(mod\;p\right)$. The prover publishes the vector $s\in Z_{p}^{n-k}$ as the public key.

### 3.2. Detailed Steps of the Lee–Stern Zero-Knowledge Identification Protocol

The prover (P) attempts to prove to the verifier (V) that he possesses a legitimate secret key e (i.e., satisfying $He^{T}=s$ and $wt_{L}\left(e\right)=w$) without disclosing any other information about e. The single-round interaction steps are as follows:

#### 3.2.1. Commitment

- First: The prover (P) randomly selects a position permutation σ from the symmetric group $S_{n}$ degree n.
- Second: The prover (P) randomly selects a mask vector $u\in Z_{p}^{n}$, and uniformly generates three independent random salts $\rho_{1},\rho_{2},\rho_{3}\in {\left\{0,1\right\}}^{\lambda }$ (where λ is the security parameter, e.g., 256 bits).
- Third: The prover (P) calculates the following three commitment values $c_{1},c_{2},c_{3}$:

(4) $$c_{1}=Hash\left(\rho_{1}∥\sigma ∥\left(H\cdot u^{T}\right)\right)$$

(5) $$c_{2}=Hash\left(\rho_{2}∥\sigma \left(u\right)\right)$$

(6) $$c_{3}=Hash\left(\rho_{3}∥\sigma \left(e+u\right)\right)$$

- (Note: ∥ denotes string concatenation; $\sigma \left(x\right)$ denotes the permutation operation on vector x)
- Fourth: The prover (P) sends the commitments $c_{1},c_{2},c_{3}$ to the verifier.

#### 3.2.2. Challenge

The verifier (V) randomly and uniformly selects a challenge factor $b\in \left\{0,1,2\right\}$ and sends it to the prover (P).

#### 3.2.3. Response and Verification

The prover generates a response according to the received challenge factor b, and the verifier performs consistency checks accordingly:

- When b=0:

Response: P reveals the permutation σ, the mask u, and the salts $\rho_{1},\rho_{2}$.

Verification: V calculates $c_{1}^{\prime }=Hash\left(\rho_{1}∥\sigma ∥\left(H\cdot u^{T}\right)\right)$ and $c_{2}^{\prime }=Hash\left(\rho_{2}∥\sigma \left(u\right)\right)$, and verifies whether $c_{1}^{\prime }=c_{1}$ and $c_{2}^{\prime }=c_{2}$ hold.

- When b=1:

Response: P reveals the permutation σ, the blinded vector $z=e+u\;\left(mod\;p\right)$, and the salts $\rho_{1},\rho_{3}$.

Verification: V independently calculates $c_{1}^{\prime }$ and $c_{3}^{\prime }$.

Since $H\cdot z^{T}=H\cdot {\left(e+u\right)}^{T}=H\cdot e^{T}+H\cdot u^{T}=s^{T}+H\cdot u^{T}\;\left(mod\;p\right)$.

Therefore, the syndrome of the mask can be derived: $H\cdot u^{T}=H\cdot z^{T}-s^{T}\;\left(mod\;p\right)$.

V calculates $c_{1}^{\prime }=Hash\left(\rho_{1}∥\sigma ∥\left(H\cdot z^{T}-s^{T}\right)\right)$.

V calculates $c_{3}^{\prime }=Hash\left(\rho_{3}∥\sigma \left(z\right)\right)$.

Verify whether $c_{1}^{\prime }=c_{1}$ and $c_{3}^{\prime }=c_{3}$ hold.

- When b=2:

Response: P reveals the permuted secret vector $v=\sigma \left(e\right)$, the permuted mask vector $w^{\prime }=\sigma \left(u\right)$, and the salts $\rho_{2},\rho_{3}$.

Verification: V performs the following three checks:

Verify whether the vector v strictly belongs to the predefined composition set $S_{T}$ (i.e., verifying that v has the exact multiset of Lee values defined by T).

Calculate $c_{2}^{\prime }=Hash\left(\rho_{2}∥w^{\prime }\right)$, and verify whether $c_{2}^{\prime }=c_{2}$ holds.

Calculate $c_{3}^{\prime }=Hash\left(\rho_{3}∥\left(v+w^{\prime }\right)\;\left(mod\;p\right)\right)$, and verify whether $c_{3}^{\prime }=c_{3}$ holds.

To reduce the single-round cheating probability (approximately $\frac{2}{3}$) to a negligible security level (e.g., $2^{-128}$), the above three-round interaction process needs to be independently repeated multiple times.

### 3.3. Differences from the Original Stern Zero-Knowledge Identification Protocol

In the traditional binary Hamming metric, to improve system security, usually only the code length n can be simply increased. Meanwhile, in the Lee metric (with field size p), the attacker’s search space depends not only on the positions of non-zero elements but also on the size of each element, i.e., the offset. Therefore, the Lee metric allows us to exponentially improve security by moderately increasing the prime p while maintaining a small code length n. Although this protocol follows the design idea of the classic Stern zero-knowledge identification protocol in the macro “commitment–challenge–response” architecture, it has undergone fundamental changes in the underlying algebraic structure and hard problem assumptions. The main differences are reflected in the following two aspects:

#### 3.3.1. Extension of Vector Space (From $F_{2}^{n}$ to $Z_{p}^{n}$)

The original Stern zero-knowledge identification protocol is built on the binary finite field $F_{2}$, with its vector space being $F_{2}^{n}$, and all matrix operations and vector additions are performed under the modulo 2 arithmetic [3]. The Lee–Stern zero-knowledge identification protocol proposed in this paper extends the underlying algebraic structure to the prime modulus integer finite field $Z_{p}^{N}$ (where p is a prime greater than 2) [5]. This extension brings two significant advantages:

- Richer algebraic structure. By increasing p, the solution space of the protocol can be greatly expanded without significantly increasing computational overhead, thereby effectively resisting specific types of algebraic structure attacks.
- Better flexibility. The size of the field p can be used as an independent adjustment parameter. In actual cryptosystem design, p can be adjusted to adapt to different security scenarios and communication bandwidth constraints while fixing the code length n.

#### 3.3.2. Conversion of Weight Calculation Metric (From Hamming to Lee)

This is the most core difference between this protocol and the classic Stern zero-knowledge identification protocol.

- Hamming metric. In the original protocol, the weight of the error vector is measured by Hamming distance, i.e., $wt_{H}\left(x\right)$ only focusing on the number of non-zero elements in the vector. Its underlying NP-Hard problem is the traditional SD problem in the Hamming metric.
- Lee metric. This protocol adopts Lee weight for measurement. For a vector $x\in Z_{p}^{n}$, its Lee weight is defined as $wt_{L}\left(x\right)=\sum_{i=1}^{n}\min\left(x_{i},p-x_{i}\right)$. The Lee metric not only focuses on whether an element is non-zero but also on the size of the element, i.e., the offset. This feature makes it have better cryptographic applicability in special physical scenarios such as phase modulation channels. At this time, the underlying security of the protocol is reduced to the SD problem in the Lee metric.

## 4. Security Analysis

This chapter will conduct a comprehensive theoretical proof and anti-attack evaluation on the security of the Lee–Stern zero-knowledge identification protocol. We will first prove that it satisfies the three core properties of zero-knowledge identification protocols: completeness, soundness and zero-knowledge. Subsequently, combined with the latest LMMT-ISD attack algorithm, we will accurately reconstruct the security parameters of the protocol in the post-quantum era.

### 4.1. Theoretical Security Proof

#### 4.1.1. Completeness

Completeness means that if the prover is honest (i.e., indeed masters the secret vector e satisfying $He^{T}=s\mathrm{mod}p$ and $wt_{L}\left(e\right)=w$) and strictly follows the protocol steps, the verifier will necessarily accept his proof with 100% probability [12].

We derive the three challenge cases of the verifier respectively:

- When b=0: The prover reveals σ and u. The $c_{1}^{\prime }=Hash\left(\sigma ∥\left(H\cdot u^{T}\right)\right)$ and $c_{2}^{\prime }=Hash\left(\sigma \left(u\right)\right)$ independently calculated by the verifier are obviously completely consistent with the commitments sent by the prover in the first round, and the verification passes.
- When b=1: The prover reveals σ and z=e+u.

For $c_{1}^{\prime }$: Since $H\cdot z^{T}=H\cdot (e+u{)}^{T}=H\cdot e^{T}+H\cdot u^{T}=s^{T}+H\cdot u^{T}$, the verifier computes $H\cdot z^{T}-s^{T}=H\cdot u^{T}$. Therefore, the computed $c_{1}^{\prime }$ is necessarily equal to $c_{1}$.

For $c_{3}^{\prime }$: The verifier calculates $c_{3}^{\prime }=Hash\left(\sigma \left(z\right)\right)$, which is completely consistent with the logic of generating $c_{3}$. The verification passes.

- When b=2: The prover reveals $v=\sigma \left(e\right)$ and $w^{\prime }=\sigma \left(u\right)$. Since position permutation σ does not change the overall Lee weight of the vector, $wt_{L}\left(v\right)=wt_{L}\left(\sigma \left(e\right)\right)=wt_{L}\left(e\right)=w$ necessarily holds, and the verification passes.

The verifier calculates $c_{2}^{\prime }=Hash\left(w^{\prime }\right)=Hash\left(\sigma \left(u\right)\right)=c_{2}$;

Calculates $c_{3}^{\prime }=Hash\left(v+w^{\prime }\right)=Hash\left(\sigma \left(e\right)+\sigma \left(u\right)\right)=Hash\left(\sigma \left(e+u\right)\right)=c_{3}$. The verification passes.

In summary, an honest prover can always pass the verification.

#### 4.1.2. Soundness

Soundness means that if the prover is malicious (i.e., does not master a legitimate secret vector e), the probability that he can cheat the verifier and pass the test smoothly is extremely low [12].

Assume there exists a malicious prover who, in order to pass the verification under any challenge $b\in \left\{0,1,2\right\}$, must be able to simultaneously give legitimate responses for all three cases. If he can do this, according to the logic of the knowledge extractor, as long as the hash function is collision-resistant, we can extract the solution to the L-SDP hard problem from the three legitimate responses of the cheater [3]:

- From the successful response of b=0, the extractor obtains $\sigma_{0}$ and $u_{0}$, satisfying $c_{1}=Hash\left(\sigma_{0}∥\left(H\cdot u_{0}^{T}\right)\right)$ and $c_{2}=Hash\left(\sigma_{0}\left(u_{0}\right)\right)$.
- From the successful response of b=1, the extractor obtains $\sigma_{1}$ and $z_{1}$, satisfying $c_{1}=Hash\left(\sigma_{1}∥\left(H\cdot z_{1}^{T}-s^{T}\right)\right)$ and $c_{3}=Hash\left(\sigma_{1}\left(z_{1}\right)\right)$.
- From the successful response of b=2, the extractor obtains $v_{2}$ and $w_{2}^{\prime }$, satisfying $wt_{L}\left(v_{2}\right)=w$, $c_{2}=Hash\left(w_{2}^{\prime }\right)$ and $c_{3}=Hash\left(v_{2}+w_{2}^{\prime }\right)$.

Due to collision resistance of the hash function, the following inferences can be drawn from the above equality of commitments:

- From the equality of $c_{1}$: $\sigma_{0}=\sigma_{1}$ (denoted as σ), and $H\cdot u_{0}^{T}=H\cdot z_{1}^{T}-s^{T}$.
- From the equality of $c_{2}$: $w_{2}^{\prime }=\sigma_{0}\left(u_{0}\right)=\sigma \left(u_{0}\right)$.
- From the equality of $c_{3}$: $\sigma_{1}\left(z_{1}\right)=v_{2}+w_{2}^{\prime }$, substituting into the above formula gives $\sigma \left(z_{1}\right)=v_{2}+\sigma \left(u_{0}\right)$. Since permutation is a linear operation, transposing gives $v_{2}=\sigma \left(z_{1}-u_{0}\right)$.

At this point, the extractor constructs the vector $e^{*}=z_{1}-u_{0}$. We verify whether $e^{*}$ is a legitimate solution to the L-SDP:

- Check equation: From inference 1 above $H\cdot z_{1}^{T}-s^{T}=H\cdot u_{0}^{T}$, transposing gives $H\cdot (z_{1}-u_{0}{)}^{T}=s^{T}$, i.e., $H\cdot (e^{*}{)}^{T}=s^{T}$.
- Weight bound: From inference 3 above, $\sigma \left(e^{*}\right)=v_{2}$. Since $wt_{L}\left(v_{2}\right)=w$ is known and permutation does not change weight, $wt_{L}\left(e^{*}\right)=w$.

This means that if a malicious prover can simultaneously respond to all three challenges, the extractor can find the solution $e^{*}$ to the L-SDP problem in polynomial time. It is known that L-SDP is an NP-complete problem, so such a cheater cannot exist in polynomial time. A malicious prover can only prepare at most 2 legitimate responses in advance in a single round, and the upper bound of the probability of successful cheating in a single round is strictly constrained to $\frac{2}{3}$. Repeating N rounds, the cheating probability drops to $(2/3{)}^{N}$.

#### 4.1.3. Zero-Knowledge

Zero-knowledge requires that the verifier cannot obtain any additional information about e except the fact that “the prover indeed possesses the secret e”. To prove zero-knowledge, we construct a polynomial-time simulator that does not know the secret key e but can output a transcript indistinguishable from a real interaction.

A crucial flaw in naive adaptations to the Lee metric is that revealing $\sigma \left(e\right)$ leaks the exact multiset of the entry values under the Lee metric, which cannot be accurately simulated by merely sampling a random vector of weight w. By strictly restricting the secret key to a public fixed composition set $S_{T}$, we perfectly resolve this issue. The simulator operates by guessing the challenge $b^{\prime }\in \left\{0,1,2\right\}$:

**If guessing** $b^{\prime }=0$**:** The simulator randomly generates σ, u, $\rho_{1}$, and $\rho_{2}$. It calculates $c_{1}$ and $c_{2}$ honestly, and generates a random string of the same length as the hash output as $c_{3}$. If the verifier actually sends b=0, the simulator outputs $\sigma ,u,\rho_{1},\rho_{2}$.

**If guessing** $b^{\prime }=1$**:** The simulator randomly generates σ, a blinded vector z, $\rho_{1}$, and $\rho_{3}$. It calculates $c_{1}=Hash\left(\rho_{1}∥\sigma ∥\left(H\cdot z^{T}-s^{T}\right)\right)$ and $c_{3}=Hash\left(\rho_{3}∥\sigma \left(z\right)\right)$, and generates a random string for $c_{2}$. If b=1 is received, the simulator outputs $\sigma ,z,\rho_{1},\rho_{3}$.

**If guessing** $b^{\prime }=2$**:** The simulator randomly samples a vector $\tilde{v}$ uniformly from the fixed composition set $S_{T}$. It then samples a random vector ${\tilde{w}}^{\prime }\in Z_{p}^{n}$, and generates $\rho_{2},\rho_{3}$. It computes $c_{2}=Hash\left(\rho_{2}∥\tilde{w}\prime \right)$ and $c_{3}=Hash\left(\rho_{3}∥\left(\tilde{v}+\tilde{w}\prime \right)\;\left(mod\;p\right)\right)$, generating a random string for $c_{1}$. If b=2 is received, it outputs $\tilde{v},{\tilde{w}}^{\prime },\rho_{2},\rho_{3}$.

Because both the real prover’s $v=\sigma \left(e\right)$ and the simulator’s $\tilde{v}$ are uniformly distributed over the exact same set $S_{T}$ (since applying a random permutation σ to $e\in S_{T}$ yields a uniformly random element in $S_{T}$), the real transcript distribution and the simulated transcript distribution are statistically identical.

**Remark on Hardness Assumption:** It is important to clarify the nature of the underlying hard problem. While the general syndrome decoding problem in the Lee Metric (L-SDP) is theoretically proven to be NP-complete (which primarily indicates worst-case hardness), the cryptographic soundness of our protocol relies on its average-case hardness. Specifically, we assume that for a uniformly random parity-check matrix H and a secret vector e sampled uniformly from the fixed composition set $S_{T}$ at or near the Lee-metric Gilbert–Varshamov (GV) bound, recovering the error vector e from the syndrome s is computationally intractable in the average case. This aligns with the standard cryptographic assumptions universally adopted in code-based cryptography.

### 4.2. Anti-Attack Evaluation Based on LMMT-ISD

The security of the Lee–Stern zero-knowledge identification protocol is based on the syndrome decoding problem in the Lee metric (L-SDP). Information Set Decoding (ISD) algorithms are currently the most effective means for solving this problem.

**Domain Clarification and Lee-GV Bound.** The state-of-the-art LMMT-ISD algorithm proposed by Li and Wang [7] was originally formalized over Galois rings $Z_{p^{m}}$. In our protocol, we set m=1, which naturally degenerates the domain to the prime field $Z_{p}$, perfectly aligning with our base algebraic structure. To ensure maximum resistance against ISD algorithms, the target composition T (and thus the total weight w) must be chosen close to the Lee-metric Gilbert–Varshamov (GV) bound. Let $V_{L}\left(n,w,p\right)$ denote the volume of a Lee sphere of radius w. The GV bound is the weight $w_{GV}$ satisfying $V_{L}\left(n,w_{GV},p\right)\approx p^{n-k}$. Operating at this boundary maximizes the average-case hardness, as the expected number of solutions to the syndrome equation is exactly one.

**Concrete Quantum Complexity.** Relying on the asymptotic formula $C_{ISD}=p^{c+o\left(1\right)n}$ (as in Equation (8)) is insufficient for evaluating modern post-quantum security because it ignores polynomial factors and quantum speedups. Let $C_{iter}$ be the classical computational cost per iteration of the LMMT-ISD algorithm, and $P_{succ}$ be the success probability of a single iteration. Classically, the total complexity is $C_{classical}=\frac{C_{iter}}{P_{succ}}$.

In the post-quantum setting, an adversary can apply Grover’s amplitude amplification to the outer loop of the ISD framework. This quadratically reduces the required number of iterations from $\frac{1}{P_{succ}}$ to $\approx \frac{1}{\sqrt{P_{succ}}}$. Thus, the concrete quantum time complexity $C_{quantum}$ is formulated as:

(7) $$C_{quantum}=\min\left(\frac{C_{iter}}{P_{succ}},C_{iter}^{Q}\cdot \frac{1}{\sqrt{P_{succ}}}\right)$$

where $C_{iter}^{Q}$ represents the quantum gate cost for evaluating one ISD iteration.

**Revised Post-Quantum Parameters.** Taking Grover’s speedup into account, a classical complexity of $2^{142}$ degrades to approximately $2^{71}$ under quantum attacks, which falls short of the NIST Category 1 (128-bit) security level. To strictly achieve 128-bit quantum security ($C_{quantum}\geq 2^{128}$), we must scale up the dimensions. Based on the concrete quantum complexity framework, we propose the updated benchmark parameters:

**Security parameter set:** Modulus p=251, code length n=256, information bits k=128. The target composition T is selected to yield a total weight w≈370, which rigorously aligns with the Lee–GV bound for these dimensions. This parameter set ensures a minimum of 128 bits of security against fully scaled quantum computers.

### 4.3. Resistance to Machine Learning Attacks

With the proliferation of machine learning (ML) and deep learning (DL) capabilities, adversaries may attempt to utilize neural networks to reverse-engineer the challenges or approximate the L-SDP solutions. The Lee–Stern protocol is inherently resilient to such attacks for two main reasons. First, the protocol leverages a cryptographically secure hash function (e.g., SHA-256) modeled as a random oracle for the commitments. The highly non-linear, avalanche effect of these hash functions effectively nullifies gradient-based DL attacks, as the entropy of the output is cryptographically smooth and devoid of recognizable patterns. Second, the underlying L-SDP is a high-dimensional lattice/coding problem. Current empirical studies indicate that while neural networks can decode some short or structured error-correcting codes, they completely fail to generalize over large, random linear codes (where n≥200) at the GV bound, as required by our parameter set. Consequently, ML/DL-based adversaries cannot achieve a polynomial-time advantage over combinatorial ISD algorithms.

## 5. Experimental Evaluation

### 5.1. Theoretical Complexity Analysis

#### 5.1.1. Communication Complexity

The communication overhead of the Lee–Stern protocol mainly consists of the hash commitments in the first round and the response data in the third round. Let $L_{h}$ be the output bit-length of the hash function (in this paper, SHA-256 is adopted, i.e., $L_{h}=256$ bits).

In the first round, the prover fixedly sends three commitments, totaling $3L_{h}$ bits. The response length in the third round depends on the challenge factor $b\in \left\{0,1,2\right\}$:

- When b=0 or b=1: The prover sends a permutation $\sigma \in S_{n}$ and a vector in $Z_{p}^{n}$. Encoding the permutation optimally requires $\left\lceil n\log_{2}n\right\rceil$ bits, and encoding the vector requires $n\left\lceil \log_{2}p\right\rceil$ bits. (In our updated protocol, the 256-bit random salts are also revealed, adding a constant overhead).
- When b=2: The prover sends two permuted vectors in $Z_{p}^{n}$, requiring $2n\left\lceil \log_{2}p\right\rceil$ bits, plus the random salts.

Ignoring the constant size of the random salts for asymptotic analysis, the exact maximum communication bits per round $C_{comm}$ is strictly formulated as:

(8) $$C_{comm}=3L_{h}+\max\left(\left\lceil n\log_{2}n\right\rceil +n\left\lceil \log_{2}p\right\rceil ,2n\left\lceil \log_{2}p\right\rceil \right)\mathrm{bits}$$

This theoretical formula clearly points out that the communication overhead increases approximately as $O\left(n\log n+n\log p\right)$.

#### 5.1.2. Computational Complexity

The computational overhead of the protocol mainly comes from matrix–vector multiplication operations over finite fields $Z_{p}$, vector component permutations, and the evaluation process of cryptographic hash functions. Among them, the core bottleneck that accounts for the largest proportion of time are matrix multiplication.

The core bottleneck on the prover side lies in calculating $Hu^{T}\mathrm{mod}p$ when generating commitments in the first round $c_{2}$. Since the dimension of the public parity-check matrix H is $\left(n-k\right)\times n$, this matrix multiplication requires performing $n\left(n-k\right)$ modular multiplication and modular addition operations over $Z_{p}$.

The core bottleneck on the verifier side lies in calculating H for consistency check when the challenge is b=1, and its calculation scale is exactly the same as that of the prover.

Let the code rate of the system $R=\frac{k}{n}$ be a constant (default to R=0.5 in the experiments of this paper), then the maximum theoretical computational complexity per round of interaction $C_{comp}$ (counted by the number of basic operations over finite fields) can be expressed as:

(9) $$C_{comp}=O\left(n\left(n-k\right)\right)=O\left(n^{2}\left(1-R\right)\right)=O\left(n^{2}\right)$$

This theoretically demonstrates two important performance characteristics: first, the execution time of the protocol is extremely sensitive to the code length n, showing a strict quadratic $O\left(n^{2}\right)$ growth trend; second, since the numerical size of the prime p has an extremely weak impact on the instruction clock cycle of modern CPUs executing a single basic algebraic operation, the overall time is theoretically insensitive to p.

### 5.2. Experimental Setup for Performance Scaling

To evaluate the performance scaling of the Lee–Stern protocol, we implemented a Python (Version3.13.9) prototype. Rather than directly simulating the heavy 128-bit quantum-secure parameters (n=256) defined in Section 4.2, we first conducted experiments on a set of baseline structural parameters to observe the growth trends of computational time and communication overheads. We selected three scaling parameter sets to observe their baseline costs:

- **Baseline Group 1** (p=251,n=100,k=50,w=145): Shows a baseline single-round time of ~0.55 ms and a communication volume of ~350 bytes.
- **Baseline Group 2** (p=839,n=100,k=50,w=261): With a larger field, the average single-round time slightly increases to ~0.69 ms and the communication volume increases to ~480 bytes.
- **High-Rate Control Group** (p=251,n=100,k=75,w=31): Due to the adoption of a high code rate (R=0.75), it has the shortest time (0.30 ms), but its algebraic structure provides significantly lower hardness, demonstrating the necessity of the parameter boundaries derived earlier.

### 5.3. Impact of Code Length n on Performance

**Figure 2:**
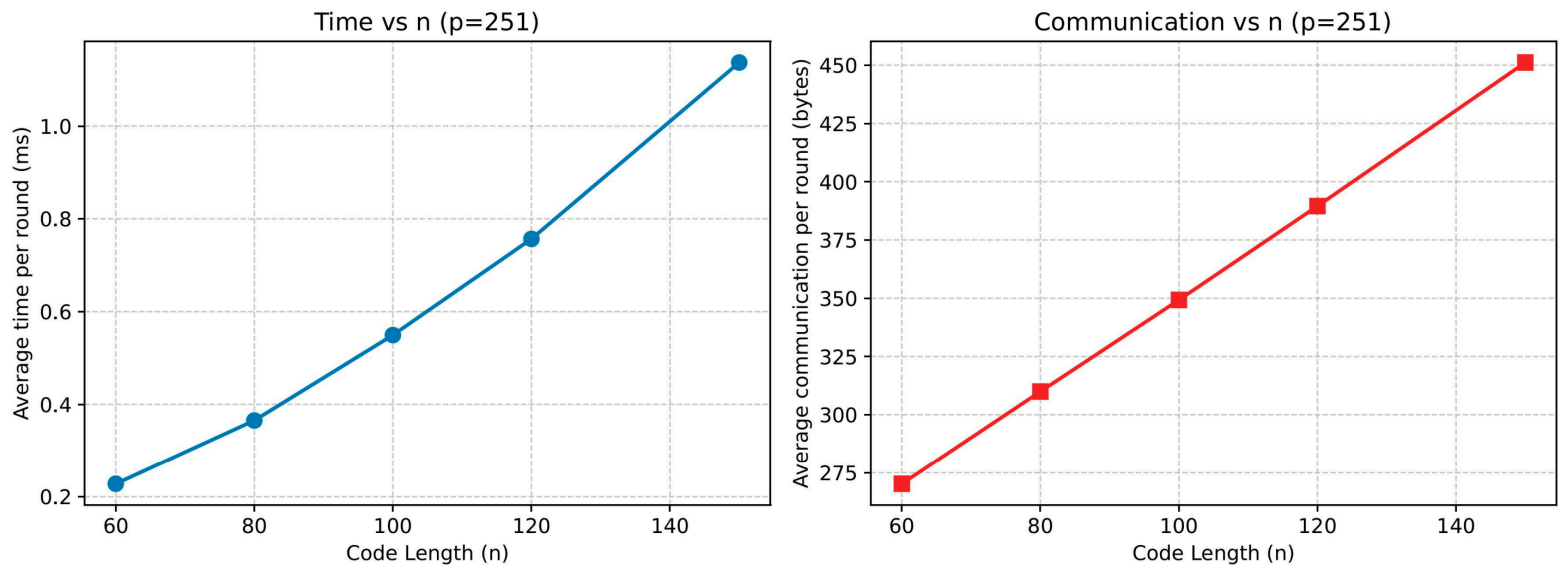
Impact of code length on performance.

Under the premise of fixed modulus p=251 and code rate R=0.5, we tested the performance changes when the code length n increases from 60 to 150 (see Figure 2).

- Computational overhead analysis: The average time increases rapidly with the increase of n, climbing from 0.21 ms (n=60) to 1.10 ms (n=150). The main computational overhead of the protocol comes from matrix multiplication (complexity about $O\left(n^{2}\right)$). The data shows that when n doubles (e.g., from 60 to 120), the time increases by about 3.3 times, which is basically consistent with the theoretical quadratic growth trend.
- Communication overhead analysis: The communication volume increases linearly with n, uniformly increasing from ~270 bytes (n=60) to ~450 bytes (n=150). This is mainly because, in addition to the fixed 96-byte hash commitment, the length of the response vector directly depends on the size of n.

### 5.4. Impact of Prime p on Performance

**Figure 3:**
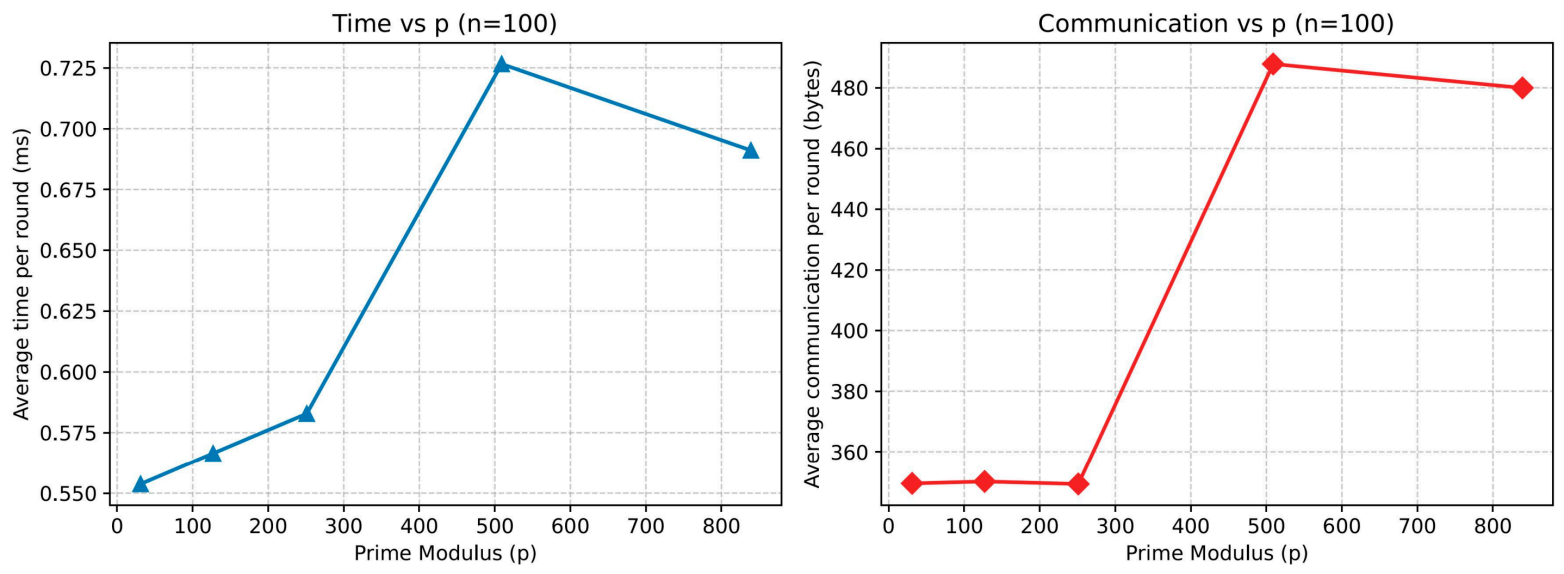
Impact of prime on performance.

Under the condition of fixed structural parameters (n=100, k=50, w=145), we investigated the impact of the prime field size p on the protocol performance from 31 to 839 (see Figure 3).

- Computational overhead analysis: The change of p has an extremely weak impact on computational overhead. Under all tested *p* values, the single-round time is basically stable between 0.52 ms and 0.68 ms. This indicates that we can introduce richer algebraic structures by increasing p without significantly increasing computational cost, which gives Lee distance great flexibility.
- Communication overhead analysis: The communication volume shows a stepwise change. When p increases from 31 to 251, the communication volume is stable at about ~350 bytes; whereas when p exceeds 255, the communication volume surges to ~480 bytes. This is because when the computer encodes and stores vector components, elements within p=251 can be represented by 1 byte, while larger primes require 2 bytes for storage.

### 5.5. Horizontal Comparison with the Original Scheme

To demonstrate the practical performance of the Lee–Stern protocol, we compare it horizontally with the classic binary Stern protocol (and its variants). To ensure absolute fairness, both schemes are scaled to provide a strict 128-bit **quantum** security level. Achieving this in the binary Hamming metric typically requires n≈700 and k≈350. For our Lee–Stern protocol, the dimensions are set to n=256,k=128,p=251.

Furthermore, because the single-round cheating probability of the Stern-type protocol is $\frac{2}{3}$, both protocols must be repeated independently N times to achieve the standard $2^{-128}$ soundness error. The required number of rounds is $N=\left\lceil -\frac{128}{\log_{2}\left(\frac{2}{3}\right)}\right\rceil \approx 219$ rounds.

The specific performance index comparison is summarized in Table 1.

Analysis of Comparative Advantages. Reviewing the rigorously calculated data in Table 1 yields the following insights:

- **Accurate Public Key Cost.** The actual per-user public key is the syndrome vector s, not the system-wide parity-check matrix H. While the Lee–Stern protocol expands the underlying field (requiring about 1 byte per element), its actual public key size remains highly compact at ~128 Bytes. This is slightly larger than the binary protocol’s ~44 Bytes but still orders of magnitude smaller than lattice-based signatures like Dilithium.
- **Reduction in Code Length.** By utilizing the “offset size” inherent in the Lee metric alongside error positioning, we condense the required code length from 700 down to 256.
- **Total Multi-Round Efficiency.** For practical deployment, a zero-knowledge identification protocol must execute ~219 rounds. Our protocol cuts the total communication volume by more than half (from ~240 KB down to ~112 KB) compared to the classical binary scheme, making it substantially more bandwidth-efficient for post-quantum authentication over constrained channels.

## Figures and Tables

**Table 1:** Performance comparison of different identity authentication schemes at the same security level (128-bit).

Metric	Classic Binary Stern	Lee–Stern Protocol
Algebraic Structure	$F_{2}$	$Z_{251}$
Code Length	~700	256
Information Bits (k)	~350	128
Actual Public Key Size (Syndrome s)	~44 Bytes	~128 Bytes
Single-Round Comm.	~1.1 KB	~512 Bytes
Total Comm. (219 Rounds)	~240.9 KB	~112.1 KB

## Data Availability

The original contributions presented in this study are included in the article. Further inquiries can be directed to the corresponding author.
